# Regulating exopolysaccharide gene wcaF allows control of *Escherichia coli* biofilm formation

**DOI:** 10.1038/s41598-018-31161-7

**Published:** 2018-09-03

**Authors:** Jingyun Zhang, Chueh Loo Poh

**Affiliations:** 1 Department of Biomedical Engineering, Faculty of Engineering, National University of Singapore, 117583 4 Engineering Drive 3, Singapore, Singapore; 2 NUS Synthetic Biology for Clinical and Technological Innovation (SynCTI), Life Sciences Institute, National University of Singapore, 117456 28 Medical Drive, Singapore, Singapore

## Abstract

While biofilms are known to cause problems in many areas of human health and the industry, biofilms are important in a number of engineering applications including wastewater management, bioremediation, and bioproduction of valuable chemicals. However, excessive biofilm growth remains a key challenge in the use of biofilms in these applications. As certain amount of biofilm growth is required for efficient use of biofilms, the ability to control and maintain biofilms at desired thickness is vital. To this end, we developed synthetic gene circuits to control *E*. *coli* MG1655 biofilm formation by using CRISPRi/dCas9 to regulate a gene (wcaF) involved in the synthesis of colanic acid (CA), a key polysaccharide in *E*. *coli* biofilm extracellular polymeric substance (EPS). We showed that the biofilm formation was inhibited when wcaF was repressed and the biofilms could be maintained at a different thickness over a period of time. We also demonstrated that it is also possible to control the biofilm thickness spatially by inhibiting wcaF gene using a genetic light switch. The results demonstrate that the approach has great potential as a new means to control and maintain biofilm thickness in biofilm related applications.

## Introduction

Biofilms are widely found in nature and they are commonly formed by group of microorganisms sticking onto surfaces and forming slimy extracellular matrix. Biofilms are known to cause problems in many areas of human health^1^ and the industry^2,3^, including food, marine, and environment. However, by exploiting the biofilms’ unique characteristics of being tolerant and persistent in harsh environments, biofilms have also been found to be beneficial in a number of applications including wastewater management^4,5^, bioremediation^6^, continuous bioproduction of valuable chemicals^7–9^ and production of biomaterials^10–12^. Biofilms are known to have high tolerance against low pH, toxicities and antimicrobial agents which makes it useful for processing wastes and bioproduction of chemicals that are toxic to the host cells (e.g., succinic acid^8^ and ethanol^13^). Biofilms are also able to achieve high cell density and cells in biofilm state usually have higher productivity compared to planktonic cells^14^. Together with the property that the biomass can be retained during the changing of medium, biofilms are attractive as a means for continuous bioproduction^15,16^.

A common and important challenge in utilising biofilm in these applications is the excessive growth of biofilms and associated extracellular polymeric substances (EPS), causing clogging, biofouling and loss in productivity^7,15,17,18^. Excessive growth of biofilms and associated EPS would also greatly limit the diffusion of substrates and nutrients to the cells^19,20^. Consequently, the cells in the inner layers of biofilm would become less active. For the use of biofilms in wastewater treatment, the thickness of biofilm has shown to influence pollutant removal efficiency^16,21,22^. Pollutant removal efficiency of biofilm increases within certain thickness due to increased biomass, but starts to decrease as the biofilm becomes thicker because of the diffusion limitation^21,22^. In bioproduction, excess growth in biofilm limits the feed availability, thus reducing the bioreactor efficiency and productivity^15,18^. Hence, to enable more efficient use of biofilm, there is a need to be able to effectively control the growth of biofilm and maintain the thickness of the biofilm.

Due to the problems caused by biofilms, most of the previous studies have focused on either eradicating biofilms or preventing biofilm formation. These biofilm prevention/removal methods include: (i) mechanical methods (e.g., mechanical scraping; bubbling and vibration); (ii) chemical methods (e.g., the use of surfactant such as δ-hemolysin to remove biofilm^23^ and the use of nitric oxide which can lead to biofilm dispersal^24^); and (iii) biological methods. A number of biological methods which target different aspects of biofilm formation have been reported^25^. These methods include using enzymes, such as proteinase and DNase to degrade the extracellular proteins and DNA respectively^25^; expressing proteins/peptides that can target second messengers (e.g. cyclic di-guanosine monophosphate (c-di-GMP)) that regulate biofilm development^26–28^, and peptide that reduces stress responsive guanosine pentaphosphate (ppGpp) concentration^29^. Other biological methods also include quorum quenching that interrupts autoinducer-mediated quorum sensing^20,30^ and controlling the production of adhesive proteins such as csgA which could inhibit initial attachment of the bacteria^12^.

These methods have been demonstrated to be able to either remove biofilm or prevent biofilm formation. Among these methods, targeting c-di-GMP and quorum quenching are most well studied. Although targeting the signaling molecules such as c-di-GMP and quorum sensing autoinducers can be effective in reducing biofilm formation, it might affect the cell’s performance in the bioprocess as c-di-GMP and autoinducers are also involved in many other cellular activity pathways^31^. Methods that inhibit cell attachment could prevent biofilm formation but they would not be suitable for the applications that require certain amount of biofilm^32^. Thus, the use of these methods is limited to achieve long term biofilm control in continuous bioprocess. Taken together, there still lack methods that could maintain the biofilm at a certain desired thickness.

In this paper, we aimed to engineer a strain of *E*. *coli* (MG1655) in which its biofilm thickness can be controlled and maintained. This is achieved by developing synthetic gene circuits coupled with CRISPRi/dCas9 to regulate a gene (wcaF) within the *E*. *coli* MG1655 genome involved in exopolysaccharide colanic acid synthesis^33^. Previous studies have shown that CA affects the formation of biofilm thickness and it does not appear to be involved in the initial attachment^34,35^. Domka *et al*. showed that genes encoded for CA synthesis were up-regulated in mature biofilms^36^, while Danese *et al*. showed that *E*. *coli* K-12 with mutated wcaF gene could only form biofilm at one or two cell layers in depth^37^. Here, we hypothesised that by directly controlling the expression of wcaF gene which is involved in the synthesis of CA we would be able to achieve a more direct control of the biofilm thickness. To this end, synthetic gene circuits controlled by chemicals or light were developed to regulate the expression of wcaF using CRISPRi/dCas9. We designed gRNA that targets the wcaF gene within the *E*. *coli* MG1655 chromosome. Potentially, other genes involved in colanic acid synthesis could also be targeted^33^. Our results showed that it is possible to control and maintain the *E*. *coli* MG1655 biofilm thickness over time by regulating the expression of wcaF gene. To the best of our knowledge, this is the first study on inhibiting wcaF gene within the CA synthesis using CRISPRi/dCas9 for the control of biofilm growth. Our approach differs from commonly studied strategy which targets global regulators such as cyclic-di-GMP and quorum sensing molecules. The presented approach has great potential as a new means to control and maintain established *E*. *coli* 1655 biofilm thickness, which would be very useful in biofilm related applications (e.g., controlling biofilm used in wastewater treatment at certain thickness to prevent clogging and potentially increase pollutant removal efficiency).

## Results

### Repressing wcaF gene by CRISPRi/dCas9 can prevent biofilm formation

**Figure 1:**
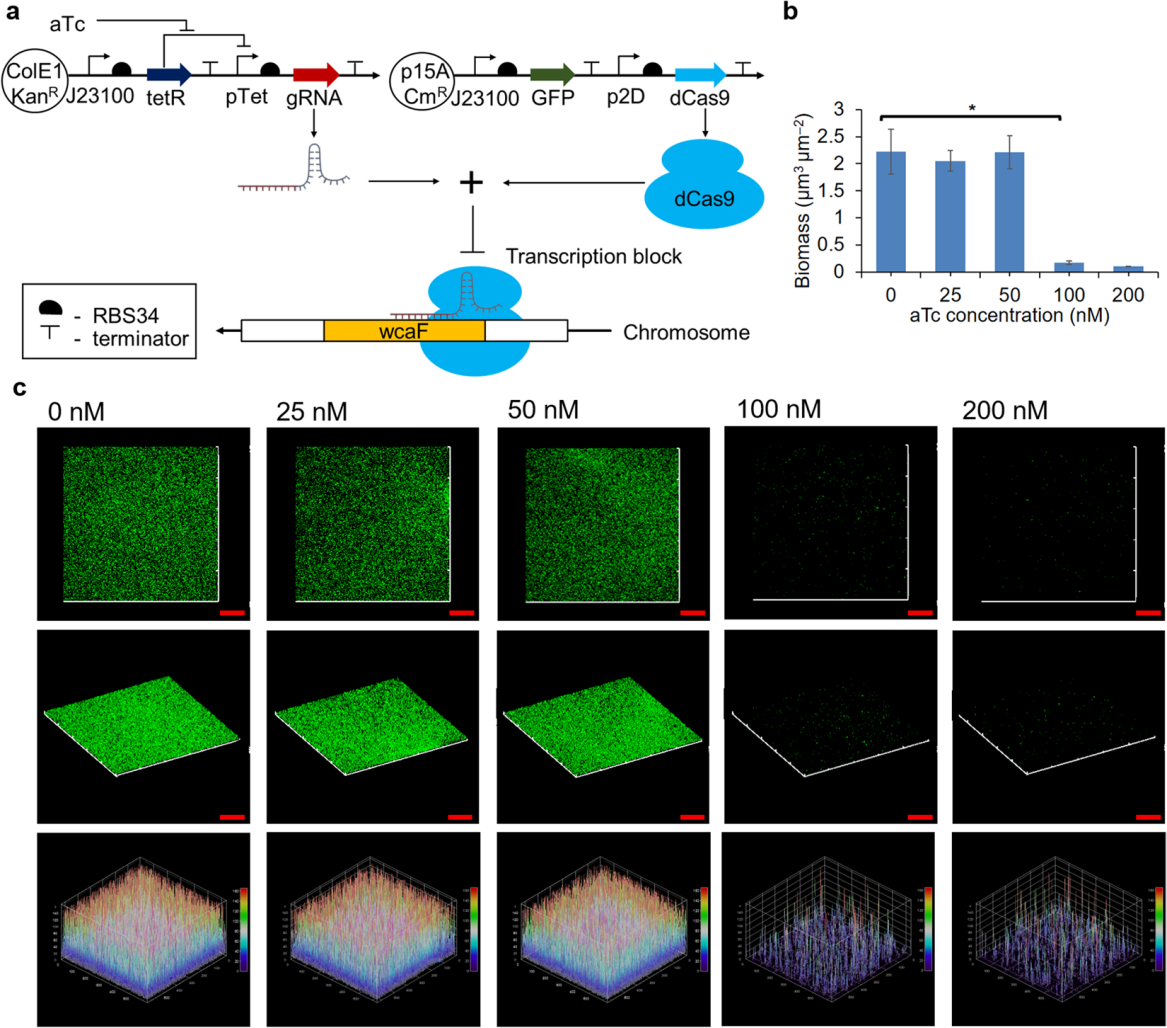
Using CRISPRi/dCas9 to repress gene (wcaF) involved in colanic acid synthesis can prevent biofilm formation. **(a)** Gene circuits that consist of constitutive dCas9 and GFP expression plasmid and aTc inducible gRNA expression plasmid. **(b)** Biomass of the biofilms derived using COMSTAT software based on confocal images. The biomass of biofilms formed by *E*. *coli* MG1655 in which the gRNA_wcaF159_ expression were induced by 25 nM and 50 nM aTc had no significant difference from the control group (gRNA−) which did not have aTc added (0 nM). In contrast, the biofilms of *E*. *coli* MG1655 in which the gRNA_wcaF159_ expression were induced by 100 nM and 200 nM aTc had significant lesser biomass than the control group (gRNA−). **(c)** Confocal images of biofilms formed by *E*. *coli* MG1655 when gRNA expression was induced by various aTc concentration. Biofilms of *E*. *coli* MG1655 with gRNA induced by 25 nM and 50 nM aTc had similar thickness with control (gRNA−), while those that were induced by 100 nM and 200 nM of aTc showed a reduced thickness of biofilm as compared to the control (gRNA−). The biofilm GFP intensity plots also indicate the thickness of biofilm induced by 100 nM and 200 nM of aTc were much thinner than the rest. Scale bars, 50 μm. All data are represented as mean ± std_dev (n = 3). *indicates P value < 0.05.

We first studied if biofilm formation could be inhibited when one of the colanic acid cluster genes was repressed by CRISPRi/dCas9. To this end, we designed gRNA to target the gene wcaF involved in colanic acid synthesis^37^. We designed gRNA that targets the location 159 of the wcaF gene (Supplementary Table 1). Constitutive dCas9 expression plasmid p2D-dCas9-J23101-GFP and anhydrotetracycline (aTc) inducible gRNA expression plasmid pBbE2k-pTet-gRNA_wcaF159_ (the numbers 159 refers to the location of wcaF gene targeted by gRNA) were constructed and co-transformed into *E*. *coli* MG1655 (Fig. 1a). Green fluorescence protein (GFP) was constitutively being expressed to serve as a reporter in the study of the biofilm formation.

We first studied which gRNA_wcaF159_ level is sufficient to inhibit biofilm growth. To achieve this, biofilms formed by *E*. *coli* MG1655 harboring p2D-dCas9-J23101-GFP and pBbE2k-pTet-gRNA_wcaF159_ were induced by 25 nM, 50 nM, 100 nM, and 200 nM of aTc respectively at the beginning of biofilm culturing (These concentrations of aTc generate a gradient of gene expression levels for the pTet promoter, Supplementary Fig. S1). Biofilms were cultured for 24 hours and the biofilm were imaged using a confocal laser scanning microscope (CLSM). The biomass derived using the COMSTAT software based on the confocal images show that the biofilms induced by 25 nM and 50 nM of aTc were similar to that of the control group (gRNA−). On the other hand, the biomass of the biofilms induced by 100 nM and 200 nM aTc had significant difference (~13 folds lesser) from the control group (Fig. 1b). The confocal images show that both biofilm induced by 25 nM and 50 nM aTc were still able to form three-dimensional biofilm structure similar to the control group (gRNA−), while the ones induced by 100 nM and 200 nM of aTc were not able to build the three-dimensional biofilm structure (Fig. 1c). The results show that the levels of gRNA induced by 100 nM and 200 nM of aTc can significantly prevent biofilm formation. Since gRNA induced by 100 nM aTc was sufficient to inhibit biofilm formation, 100 nM aTc was used for subsequent experiments that required gRNA induction. Furthermore, we also found that targeting wcaF gene using CRISPRi/dCas9 had minimal effect on *E*. *coli* MG1655 growth (Supplementary Fig. S2). These results indicate that using CRISPRi/dCas9 to repress wcaF gene at the beginning of biofilm culturing can significantly inhibit biofilm formation with minimal effect on the *E*. *coli* MG1655 growth.

### Repressing wcaF gene at different time point maintained *E. coli* MG1655 biofilm at different thickness

Excessive growth of biofilms could cause clogging, biofouling and loss in productivity over time^7,15,17,18^. In a separate experiment, we studied the effect of increasing biofilm thickness on the expression of red fluorescence protein (RFP) induced by chemical anhydrotetracycline (aTc). We found that the GFP and RFP expression in the biofilm differs layer by layer (Supplementary Fig. S3). This could be due the accessibility of the oxygen, nutrients and inducer at different biofilm layers. Biofilm heterogeneity was believed to be caused by the microenvironment gradient established by the local oxygen, nutrients and metabolites concentration^38^. We observed that when the biofilm grew thicker, the inner layer cells became less productive and the overall RFP saturated. Increase in biofilm biomass did not increase the overall protein expression. This suggests that it would be beneficial to maintain the thickness of biofilm at a particular thickness for biofilm related applications, instead of having biofilm that would keep growing in thickness over time.

Next, we investigated whether it is possible to maintain biofilm at different thickness by repressing wcaF gene at different time point. In our previous experiment, we found that inhibiting wcaF did not reduce biofilm thickness. Hence, we hypothesised that we could maintain established biofilm thickness by repressing wcaF gene at different time point of the biofilm formation.

**Figure 2:**
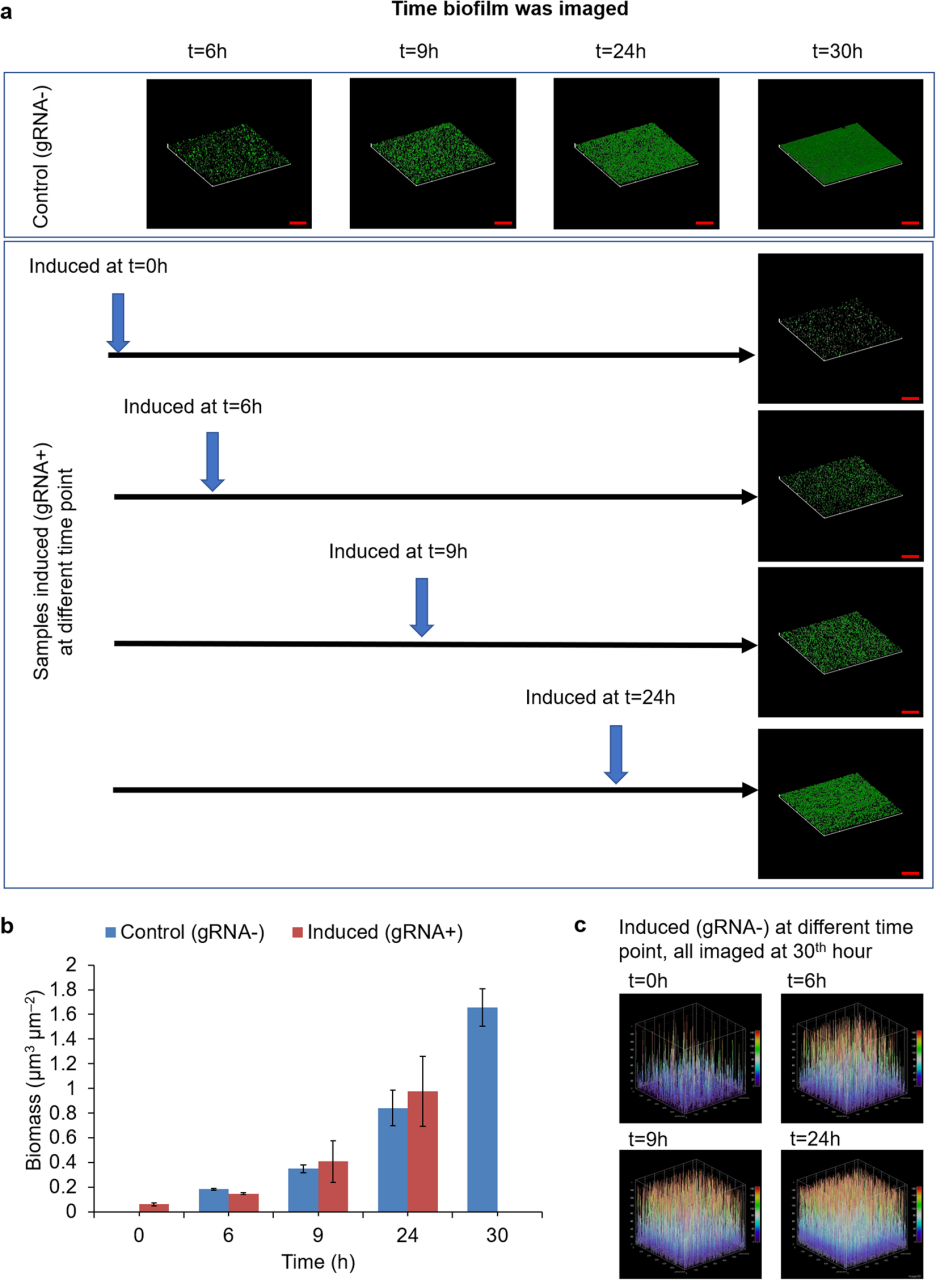
Repressing wcaF gene at different time point maintained biofilm thickness. **(a)** The confocal images show the earlier aTc was added, the thinner biofilm was formed at 30^th^ hour. Although all biofilms were cultured for 30 hours, the biofilms induced (gRNA+) with aTc at 6^th^, 9^th^ and 24^th^ hour were comparable to the controls (gRNA−) grew for 6, 9 and 24 hours respectively. **(b)** The biomass of biofilms induced at 6^th^ hour, 9^th^ hour and 24^th^ hour remained similar amount as when aTc was added. The biomass of biofilm induced at 0^th^ hour has minimal amount after culturing for 30 hours. **(c)** Three-dimensional plots based on the intensity of GFP in the biofilm. The GPF plots also show biofilms induced (gRNA+) at 6^th^, 9^th^ and 24^th^ hour were comparable to the controls (gRNA−) grew for 6, 9 and 24 hours respectively. Scale bars, 50 μm. All data are represented as mean ± std_dev.

To this end, several groups of *E*. *coli* MG1655 biofilms harboring constitutive dCas9 expression plasmid (p2D-dCas9-J23101-GFP) and aTc inducible gRNA expression plasmid (pBbE2k-pTet-gRNA_wcaF159_) were cultured. For the control groups in which aTc were not added, *E*. *coli* biofilms were grown for 6, 9, 24 or 30 hours to determine the biofilm thickness at these time points. For the experimental groups, all the biofilms were grown for 30 hours, while the aTc was added at 0, 6, 9 and 24 hours to different groups respectively (Fig. 2a). The results show that the earlier aTc was added, the thinner the biofilm was observed. When aTc was added at the beginning of culturing (time = 0 h), the amount of biofilm formed after 30 hours was very minimal. More interestingly, although all induced biofilms (gRNA+) were grown for 30 hours, the thickness and biomass of the biofilms induced with aTc at 6, 9 and 24 hours were comparable to the control biofilms (gRNA−) grew for 6, 9 and 24 hours respectively (Fig. 2a,b). In addition, the three-dimensional plots based on GFP intensity show that biofilms induced (gRNA+) at 6, 9 and 24 hours maintained at different thickness after culturing for 30 hours (Fig. 2c). The results show that it is possible to maintain biofilm of different thickness. In addition, the results further show that when the expression of wcaF gene was repressed, the thickness of the *E*. *coli* MG1655 biofilm neither increased nor decreased.

### Using blue light mediated gene circuit to regulate wcaF gene expression allows spatial control of biofilm formation

**Figure 3:**
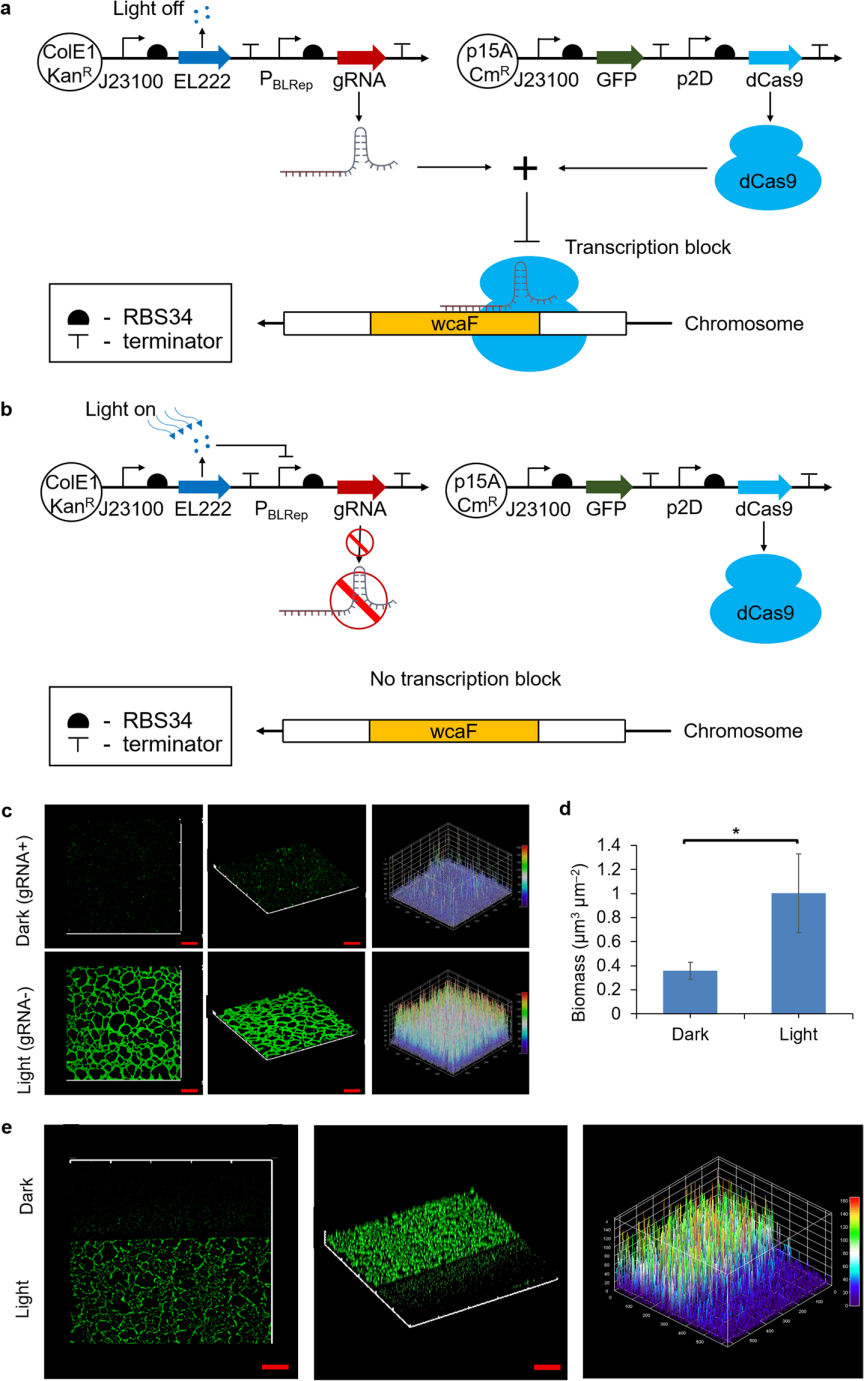
Using blue light to regulate wcaF gene expression can control biofilm thickness spatially. **(a)** Gene circuits that consist of constitutive dCas9 expression plasmid and blue light repressible gRNA expression plasmid. gRNA would be expressed and hence wcaF gene would be repressed in dark condition. **(b)** In contrast, the gRNA would be repressed and wcaF gene would be expressed when exposed to blue light. **(c)** Control biofilm thickness in spatial. Even the biofilm was cultured on the same microscope slide in the same compartment of medium, confocal images show that only the area that exposed to blue light was able to form thick biofilm. **(d)** The biomass on the region which was exposed to light was around 3 folds more than that of dark region. **(e)** At the light-dark boundary, the biofilm thickness showed difference at each side. The three-dimensional plot of GFP intensity also shows the biofilm thickness difference in spatial at the light-dark boundary. Scale bars, 50 μm. All data are represented as mean ± std_dev. *indicates P value < 0.05.

It would be useful to locally control biofilm thickness at different location on a surface. To investigate whether it is possible to spatially control the biofilm thickness through wcaF gene repression, we first explored the possibility of using light to regulate the gRNA expression which targets the wcaF gene. For this purpose, gRNA expression was put under the control of a blue light repressible promoter^39^. Plasmids Brep-gRNA_wcaF159_ and p2D-dCas9-J23101-GFP were co-transformed into *E*. *coli* MG1655. When there is no blue light, the expression of gRNA is constitutive and the expression of wcaF gene is repressed (Fig. 3a). In the presence of blue light, the expression of the gRNA will be repressed and wcaF gene will be expressed (Fig. 3b). Our hypothesis was that biofilm formation would be inhibited in the dark condition while biofilm would grow when exposed to blue light.

In this experiment, blue light was shone from the bottom of the microscope slide. As we liked to create patterns on a single slice, we designed photo mask to cover part of the biofilm growth surface (the microscope slide) so that only part of the cell on the microscope slide would be exposed to blue light (Fig. S4). Using this setup, the biofilms of *E*. *coli* MG1655 harboring plasmids Brep-gRNA_wcaF159_ and p2D-dCas9-J23101-GFP were cultured on the microscope slide for 9 hours, with the blue light being turned on throughout. Biofilms were observed under the confocal laser microscope at the 9^th^ hour of biofilm culturing. The images show that biofilm growth in the region under the dark condition (gRNA+) was inhibited, while the biofilm which was exposed to the blue light (gRNA−) formed a thicker structure (Fig. 3c). The biomass of the biofilm that was exposed to blue light was significantly more than the biofilm in the dark (Fig. 3d). Besides, it was found that across the blue-light boundary area, the biofilm formed a thick structure on the side which was exposed to blue light while biofilm did not form thick structure on the other side that was in the dark condition. The three-dimensional GFP intensity plot also shows the difference of biofilm on two sides of the light-dark boundary (Fig. 3e). The results suggest that repressing wcaF gene using CRISPRi/dCas9 has a localized effect on the biofilm formation when the biofilm was in the same culture medium compartment, allowing spatial control of biofilm formation.

### Repressing wcaF gene expression did not significantly affect biofilm tolerance against antibiotics

Biofilm tolerance to harsh conditions, such as low pH and antimicrobial agents, is a beneficial characteristic for biofilm applications in the industry (e.g., bioproduction and wastewater treatment). *E*. *coli* biofilm EPS serves as a protective layer for the cells in the biofilm. Hence, inhibiting the wcaF gene involved in the synthesis of colanic acid (a polysaccharide in the EPS) might affect the biofilm tolerance towards the harsh conditions.

To investigate if the biofilm tolerance towards the harsh conditions (e.g. the presence of antibiotics) will be affected by repressing wcaF gene, we used 25 μg/mL of erythromycin to treat the engineered biofilm and used Live/Dead Baclight assay to measure the resultant cell viability. Three groups of biofilms of *E*. *coli* MG1655 harboring plasmids p2D-dCas9-J23101-GFP and pBbE2k-pTet-gRNA_wcaF159_ were studied. For the two control groups (gRNA−), they were grown for 6 and 8 hours respectively, followed by erythromycin and Live/Dead Baclight assay treatment. For the experimental group (gRNA+), aTc was added after 6 hours to induce gRNA and repress wcaF expression. Thereafter, biofilm was cultured for another 2 hours followed by erythromycin and Live/Dead Baclight assay treatment.

**Figure 4:**
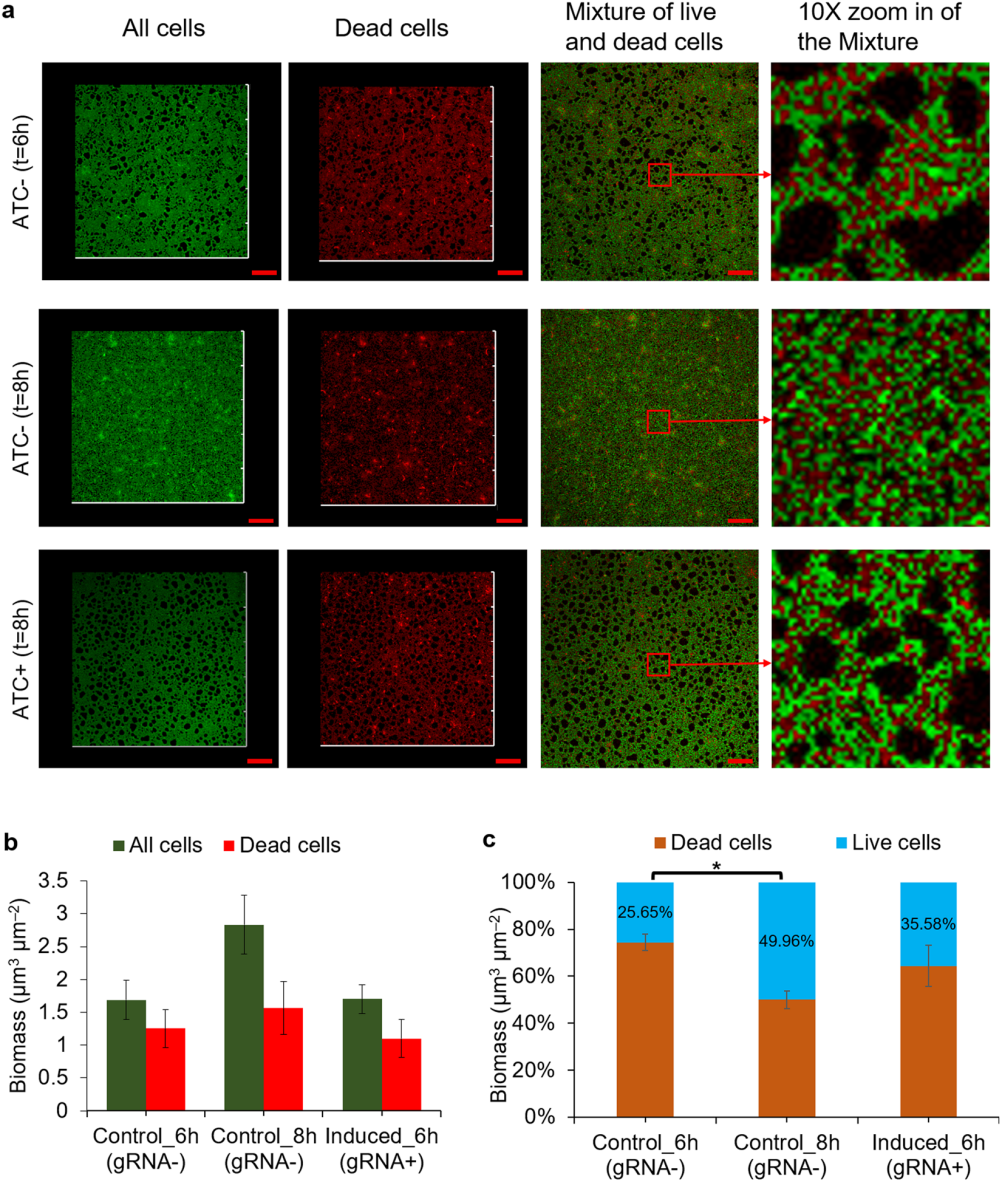
Repressing wcaF gene did not significantly affect biofilm tolerance against erythromycin. **(a)** The biofilm level of experimental group (gRNA induced at 6^th^ hour and cultured to 8^th^ hour) was similar to control group (gRNA−) grown to 6^th^ hour. After treating with erythromycin and followed by Live/Dead Baclight assay, some red fluorescence was observed, showing some cells were dead or their membranes were damaged. From the zoom in images, it can be observed that the control biofilm (gRNA−) grew to 6^th^ hour had relatively more red fluorescence as compared to the control biofilm (gRNA−) grew to 8^th^ hour. **(b)** The total biomass of experimental group (gRNA+) was similar to the one of control group (gRNA−) grown to 6^th^ hour, and they were lesser than the control group (gRNA−) grown to 8^th^ hour. **(c)** The live cells ratio of control group (gRNA−) grown to 6^th^ hour was significantly lesser than the control group (gRNA−) grown to 8^th^, which shows biofilm tolerance to erythromycin increases during biofilm developing. The live cells ratio of experimental group (gRNA−) had no significant difference from both control groups (gRNA−) that grown to 6^th^ hour. Scale bars, 50 μm. All data are represented as mean ± std_dev (n = 3). *indicates P value < 0.05.

The confocal images of the biofilms show that the experimental group (gRNA+) had similar biofilm thickness as the control group (gRNA−) grown for 6 hours, and had less biofilm as compared to the control group (gRNA−) grown for 8 hours (Fig. 4a). Biomass of total cells and dead cells between the experimental group (gRNA+) and the control group biofilm grown for 6 hours had no significant difference, but were lower than the control group (gRNA−) biofilm grown for 8 hours which had a thicker biofilm (Fig. 4b). Comparison between the two control groups (gRNA−) showed, as expected, that the one grown for 8 hours had a lower percentage of dead cells compared to the one grown for 6 hours. This suggests that the biofilm grown for 8 hours was more tolerant against the antibiotic. The control group (gRNA−) grown for 6 hours comprised the highest percentage of dead cells. The live cell ratios between the experimental group (gRNA+) and the control group (gRNA−) biofilm grown for 6 hours had no significant difference (Fig. 4c). The results imply that the tolerance of the *E*. *coli* MG1655 biofilm had not changed significantly by inhibiting wcaF expression.

## Discussion

In this paper, we have engineered *E*. *coli* MG1655 in which its wcaF gene within the genome can be regulated using aTc or light. wcaF was hypothesised to encode acetyltransferase which is involved in the synthesis of colanic acid^33^. We showed that regulating the wcaF gene affects biofilm formation. Previous studies have showed that colanic acid is essential for biofilm maturation and is related to biofilm thickness^34^. Moreover, *E*. *coli* MG1655 with mutated colanic acid cluster gene wcaF was not able to build three-dimensional biofilm structure^37^. It was hypothesised that wcaF gene is involved in the acetylation of colanic acid synthesis^33^. Besides, acetyl groups are able to increase the adhesive and cohesive properties of biofilm^40^. Unlike commonly used biofilm control methods such as using dispersin and quorum quenching which target global regulator that might have effect on many aspects of the cells, targeting wcaF gene would mainly affect colanic acid synthesis. Here, we showed that regulating wcaF gene that involved in colanic acid synthesis could potentially be an effective method to control biofilm formation, specifically maintaining established biofilm thickness.

We first investigated the use of CRISPRi to interfere the wcaF gene in the *E*. *coli* genome. wcaF gene has been suggested to be involved in colanic acid synthesis by a study involving mutations of the gene^37^. It was unclear whether directly regulating wcaF gene using CRISPRi could be used to control biofilm formation. Here, we showed that the formation of biofilm was effectively inhibited when the wcaF gene was targeted by the designed gRNA. In addition, cell growth was minimally affected when wcaF gene was repressed using the synthetic gene circuits. Consequently, this result suggests that it could be possible to maintain the biofilm at different established thickness using our approach.

By inducing gRNA at different time point, we found that the thickness of biofilms with wcaF gene repressed after 6, 8 or 24 hours, but all cultured for a total of 30 hours remain comparable with the wild type biofilms that were grown for 6, 9 and 24 hours respectively. Our study has been performed using petri dishes. It will be interesting to further study the method in a more continuous manner with the use of microfluidics^41^. Nonetheless, as a proof of concept, these results demonstrate that repressing wcaF gene could be a promising method to maintain established biofilm of different thickness. To the best of our knowledge, this is the first study to demonstrate the maintenance of biofilm thickness through the inhibition of wcaF gene using CRISPRi. This could be particularly useful for applications related to wastewater treatment and bioproduction in which the thickness of biofilm has shown to influence pollutant removal efficiency in wastewater treatment and bioproduction^15,16,18,21,22^.

We were interested to test whether we could spatially or locally control biofilm formation by regulating wcaF gene, as it would provide an added capability to control biofilm thickness at different location of a surface. Hence, we studied the expression of gRNA_wcaF_ under a blue light repressible promoter. The results showed that blue light could be used to regulate wcaF gene expression and, consequently, control biofilm formation locally. Interestingly, using light to control the expression of gRNA that regulates wcaF gene could create biofilm 2D patterning. On the same substrate surface, only the biofilm grew in the blue light shined area could form three-dimensional structure. This offers a potential means for localised control of biofilm thickness using light which could be useful in biomaterial patterning^10–12^. However, as repressing wcaF gene would not disperse biofilm, additional control such as introduction of dispersin into the gene circuit would be required for applications in which dispersal is necessary.

The EPS forms a protective layer that protects the cells within the biofilm against extreme/harsh conditions (e.g., low pH, high toxicity and antibiotics). To investigate whether the tolerance of the engineered biofilm would be significantly affected with the inhibition of wcaF gene expression, we designed experiments to test its tolerance against antibiotic, erythromycin, which would cause bacterial cell death by binding to bacterial ribosome and inhibiting protein synthesis. The tolerance of the biofilm was tested by studying the ratio of live cells against the total cells which include both live and dead cells^42^. The results show that the live cells ratio of the engineered biofilm (in which wcaF gene expression had been repressed after 6 hours) was similar with the wild type biofilm (grown for 6 hours) after treating with erythromycin. This finding suggests that inhibiting wcaF gene did not significantly reduce biofilm tolerance as compared to the biofilm grown for the same period of time. This implies that the proposed method could maintain the property needed for applications involving harsh conditions such as the production of toxic chemical and wastewater management. In this paper, we targeted wcaF gene. Other than wcaF gene, other genes involved in CA synthesis pathway, such as wzx gene which is hypothesised to participate in CA polymerization and export, gene manB and manC which are required for synthesis of nucleotide sugar precursors of CA^33^, could be potential targets to inhibit CA synthesis. Targeting CA to regulate biofilm formation could potentially be applied to other strains that also have CA, such as *Salmonella enterica* serovar Typhimurium LT2^43^. For mixed species, if this genetic modification can be used to control biofilm of each species, the mixed species biofilms could be possibly controlled by tuning the CRISPRi.

In summary, the results presented in this paper show that repressing wcaF involved in colanic acid synthesis using CRISPRi is potentially an effective means to control and maintain the thickness of biofilm, particularly for applications in wastewater treatment and bioproduction.

## Methods

### Plasmids construction

All the plasmids and primers in this project were designed *in silico* using Benchling web-based designer (Benchling, San Francisco, CA, USA). The chromosomal wcaF gene targeting gRNA was designed using the Benchling genome engineering tool (Benchling, San Francisco, CA, USA). This tool uses a model derived by Doench *et al*. for CRISPR on-targeting efficiency and a second model created by Hsu *et al*. for CRISPR off-targeting efficiency to present the user with two scores (from 1 to 100 points) for each of the gRNA^44,45^. Using the models, we chose to target location 159 of the wcaF gene coding region (see supplementary Table 1) as it gave a high score and it is near to the start codon of wcaF gene. The backbone plasmids pBbE2k, pBbE8k, and pdCas9 were supplied by Addgene (Cambridge, Massachusetts, USA)^46^. Genes and primers were obtained from using gene fragments (gBlocks) from Integrated DNA Technologies (IDT, Coralville, Iowa, United States). RFP (red fluorescence protein) and GFP (green fluorescence protein) were used as reporters for gene expression characterization and biofilm imaging. All plasmids were constructed using the Gibson assembly method^47^. All constructed plasmids were chemically transformed into *E*. *coli* MG1655 (K-12) (ATCC®700926™), which was derived from parent strain W1485 by acridine orange curing of the F plasmid. All protocols for transformations, PCR and DNA manipulation used in this work with reference to Sambrook^48^ or the manufacturer’s manual and were optimized as needed.

Glycerol stocks of all cultures were made by mixing 500 μL of the overnight culture with 500 μL of 100% glycerol and stored at −80 °C. For consistency, the overnight cultures for each experimental run were obtained by inoculating directly from their respective glycerol stocks. All bacterial cells were grown in Luria-Bertani (LB) broth medium at 37 °C with shaking speed at 225 RPM. Kanamycin (Km) (50 μg/mL) was used to maintain pBbEk-based plasmids in *E*. *coli* MG1655; Chloramphenicol (Cm) (35 μg/mL) was used to maintain pBbAc-based plasmids in *E*. *coli* MG1655. All strains and plasmids used in this study are summarized in Supplementary Table 1.

### Microplate reading to measure cell growth and RFP expression

Cells were inoculated from glycerol stock and grown for overnight in LB (50 μg/mL Kanamycin) at 37 °C with shaking speed at 225 RPM. 50ul of overnight culture was inoculated in 5 ml fresh LB (50 μg/mL Kanamycin). The cells were grown for 2 hours. OD_600_ was measured. The culture was diluted to OD_600_ of 0.1 with fresh LB (50 μg/mL Kanamycin). The inducer aTc were added to the culture to concentrations of 25 nM, 50 nM, 100 nM, and 200 nM respectively. The mixtures were then transferred into 96 well plate (Greiner Bio-One, Kremsmünster, Austria) with 300 μL in each well. Sample without aTc added served as control. Each sample has biological triplicates. The cell growth (absorbance at 600 nm) and gene expression (represented by RFP with excitation and emission wavelengths at 535 nm and 600 nm respectively) were measured using microplate reader (H1, Biotek, USA). The 96 well plate was kinetically shake, cell growth and RFP were read every 10 minutes over 8 hours. All the data was blanked with the reading of LB (50 μg/mL Kanamycin).

For measuring the growth of *E*. *coli* MG1655 harboring plasmids p2D-dCas9-J23101-GFP and pBbE2k-pTet-gRNAwcaF159 over 30 hours, cell culture was diluted to OD600 of 0.2 with fresh LB (50 μg/mL Kanamycin and 25 μg/mL chloramphenicol). The inducer aTc was added to the culture to concentrations of 50 nM, 100 nM, and 200 nM respectively. 1.5 ml of each mixture was transferred into 12 well plate (Nunc, Roskilde, Denmark). The cell growth was measured using microplate reader (H1, Biotek, USA). All the data was blanked with the reading of LB (50 μg/mL Kanamycin and 25 μg/mL chloramphenicol)

### Biofilm culturing

Biofilm was grown on the microscope slide (25 mm × 76 mm, Fisher Scientific, New Hampshire, United States) that placed in the petri dish (Greiner Bio-One, Kremsmünster, Austria). 15 ml of fresh LB supplemented with 50 μg/mL Kanamycin and 25 μg/mL Chloramphenicol, 150 μL of overnight cell culture was then added. The biofilms were grown in static culture condition in LB at 37 °C. Gently open and close the incubator door to minimize the disturbance on the biofilm.

### Biofilm characterization using confocal microscope

The microscope slide with biofilm was gently rinsed with deionized water to remove unattached cells. Microscope slide was dried at room temperature and imaged through a 20x lens with confocal laser scanning microscope (Olympus confocal FV1200, Japan). Laser wavelength of 473 nm and 559 nm were used to excite GFP and RFP respectively. Biofilm grown on the microscope slide was scanned at step size of 4 um. The three-dimensional biofilm image was obtained using the CLSM software. Image stacks of 3 random spots were collected from three sets of biofilm samples. Image stacks were saved in “tiff” format. Biomass of biofilm CLSM image was then analyzed by using the computer program COMSTAT^49–51^. Three-dimensional biofilm image based on the GFP or RFP signal intensity was plotted using the ImageJ “Stack 3D Surface Plot” function under “Plugins”^52^.

### Study heterogeneous RFP expression at different biofilm thickness

Biofilms of *E*. *coli* MG1655 harboring Constitutive GFP expression plasmid p2D-dCas9-J23101-GFP and aTc inducible RFP expression plasmid pBbE2k-pTet-RFP were cultured for 6, 9, 12, 16, 24 and 40 hours to obtain different thickness. The culture medium was changed with fresh medium at these respective time point, followed by adding 100 μM aTc and 4 hours incubation. For the control group, no aTc was added. Microscope slides with biofilm were rinsed with deionized water to remove unattached bacteria. Microscope slide was dried in room temperature overnight. Green and red fluorescence was imaged sequentially under CLSM to avoid cross-contamination of fluorescent signals. The biofilms were scanned at a step size of 2 μm. The biomass and amount of RFP at each biofilm layer were derived by COMSTAT based on the green and red fluorescence respectively.

### Blue light system to regulate wcaF gene expression

Microscope slides (25 mm × 76 mm, Fisher Scientific, New Hampshire, United States) as support for biofilm grown was illuminated using our custom-built blue light projector setup (~12 W/m^2^). Briefly, the projector has a Cree® XLamp® XP-E LED with dominating wavelength of 460 nm. The LED was powered by 1 W constant current driver module at 350 mA, stepped down from 5 V 1 A power adapter. The holder positioned the projector to get a uniform illumination on the bottom of the petri dish. For the dark condition, petri dish was wrapped in black cloth covering all edges.

Biofilm was grown on the microscope slide that was placed in the petri dish (Greiner Bio-One, Kremsmünster, Austria). 15 ml of fresh LB was supplemented with 50 μg/mL Kanamycin and 35 μg/mL Chloramphenicol, and 150 uL of overnight cell culture was then added. The biofilms were grown in static culture condition in LB at 37 °C.

For the experimental group (gRNA−), blue light projector was put under petri dish to provide blue light to the cells. Light was turned on at the beginning of biofilm culturing, so that the gRNA expression was inhibited by the blue light. For the control group (gRNA−), no blue light was put under the petri dish, and petri dish were wrapped with black cloth covering all edges. Cells in dark would have constitutive gRNA expression. Biofilms were cultured for 9 hours. Biofilm samples were collected by gently rinsing with deionized water. Samples were dried and followed by CLSM and COMSTAT processing.

### Blue light study to control biofilm thickness spatially

The above mentioned blue light system was used in this experiment. In addition, a 9 cm diameter semicircle dark paper was placed between petri dish and blue light projector to block the blue light to create dark condition for half area of the microscope slide. Blue light was turned on for 9 hours, during which half of the microscope was exposed to blue light while another half was in dark. Biofilm was cultured for 9 hours and then rinsed with deionized water to remove unattached bacteria. Microscope slide was dried in room temperature overnight. Biofilm samples were then analyzed using CLSM and COMSTAT processing.

### Biofilm tolerance test

Three sets of biofilms were cultured: (i) Control biofilm (gRNA−) cultured for 6 hours; (ii) Control biofilm (gRNA−) cultured for 8 hours; (iii) experimental biofilm with gRNA_wcaF159_ induced at 6 h and cultured for 8 hours. 100 nM aTc (Sigma, US) was used to induce gRNA_wcaF159_. To create a lethal condition for the *E*. *coli* MG1655, 25 ug/ml erythromycin was added at the time point which biofilm was stopped culturing. After treatment with 25 ug/ml erythromycin for 1 hour, biofilms were labelled using the LIVE/DEAD BacLight™ Bacterial Viability staining kit (Invitrogen, US) according to the manufacturer’s instructions. Briefly, biofilms were labelled with 1.67 μM SYTO9 (a green fluorescent dye that can cross intact membranes) and 16.8 μM propidium iodide (a red fluorescent dye that can only penetrate cells that have lost membrane integrity) at room temperature for 20 minutes in dark condition. Microscope slides with biofilm were rinsed with deionized water to remove unattached bacteria. Microscope slide was dried in room temperature overnight. Green and red fluorescence was imaged sequentially under CLSM to avoid cross-contamination of fluorescent signals. Biomass of each live and dead cells was derived by COMSTAT based on the green and red fluorescence respectively.

## Electronic supplementary material

Supplementary information

## Data Availability

All data and materials involved in this study are included in this published article and its supplementary file.
