# Supplementary material for: Regulating exopolysaccharide gene wcaF allows control of Escherichia coli biofilm formation
Source: Sci Rep. 2018 Sep 3;8:13127. doi: 10.1038/s41598-018-31161-7 (PMC6120894; doi:10.1038/s41598-018-31161-7)
Supplement: Supplementary file 1 — Supplementary information [file 41598_2018_31161_MOESM1_ESM.docx]

Supplementary information

Regulating exopolysaccharide wcaF gene allows control of *Escherichia coli* biofilm formation

Jingyun Zhang^1,2^ and Chueh Loo Poh^1,2^*

**Table of contents**

[**Figure S1: Characterization of pTet promoter using various aTc concentration. Related to Figure 1.** 1](#_Toc519289968)

[**Figure S2: Growth of E. coli MG1655 harboring constitutive dCas9 expression plasmid and aTc inducible gRNA expression plasmid at different aTc concentration. Related to Figure 1.** 2](#_Toc519289969)

[**Figure S3: Heterogenous gene (RFP) expression in the biofilm. Related to Figure 2.** 4](#_Toc519289970)

[**Figure S4: The setup used for controlling biofilm using blue light. The light system setup for spatial control of biofilm thickness. Related to Figure 3.** 5](#_Toc519289971)

[**Figure S5: Proposed pathway for CA synthesis in E. coli.** 6](#_Toc519289972)

**Figure S1: Characterization of pTet promoter using various aTc concentration. Related to Figure 1.**

pBbE2k-pTet-RFP was transformed into *E. coli* MG1655. Reporter RFP expression levels induced by aTc concentrations at 5 nM, 12.5 nM, 25 nM, 50 nM, 100 nM, and 200 nM respectively were measured. The results show that the RFP expression was almost saturated at 100 nM of aTc.


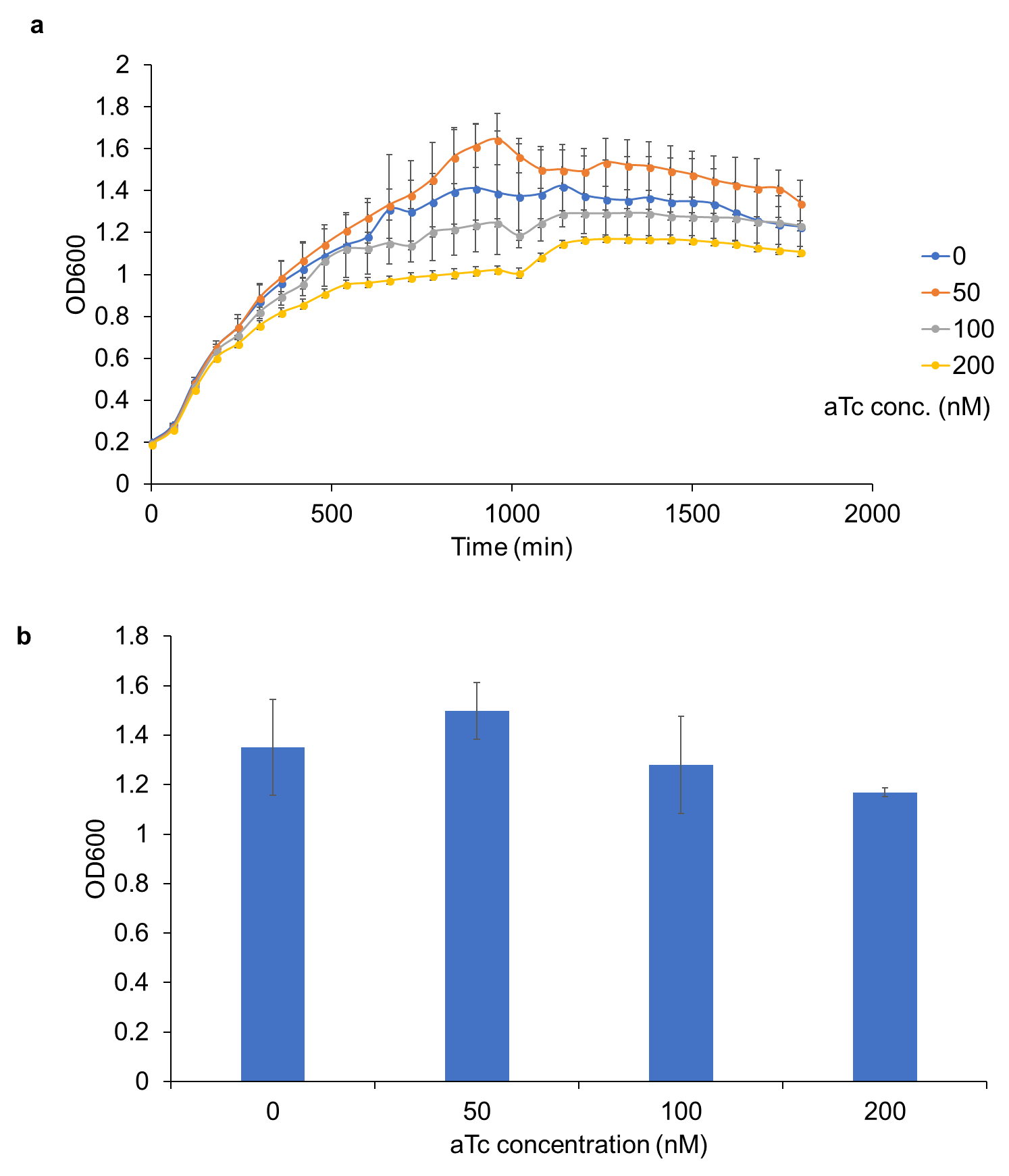


**Figure S2: Growth of E. coli MG1655 harboring constitutive dCas9 expression plasmid and aTc inducible gRNA expression plasmid at different aTc concentration. Related to Figure 1.**

Previous study has demonstrated that E. coli MG1655 defective in colonic acid synthesis was not able to form three-dimensional biofilm structure [37]. However, the effect of inhibiting wcaF gene expression on cell growth is unclear. To determine if cell growth is affected by inhibiting wcaF gene expression, the growth of E. coli MG1655 harboring plasmids p2D-dCas9-J23101-GFP and pBbE2k-pTet-gRNA_wcaF159_ was measured over 30 hours. a) Growth of the cells induced by different aTc concentration showed no difference from the growth of the control. b) OD at 24^th^ hour. The result shows using 100 nM aTc to repress wcaF gene had minimal effect on E. coli MG1655 growth. Besides, gRNA induced by 100 nM aTc was able to inhibit biofilm formation while did not affecting the growth of E. coli MG1655 harboring plasmids p2D-dCas9-J23101-GFP and aTc pBbE2k-pTet-gRNAwcaF_159_, we decided to use 100 nM aTc for subsequent gRNA induction. All data are represented as mean + std_dev.


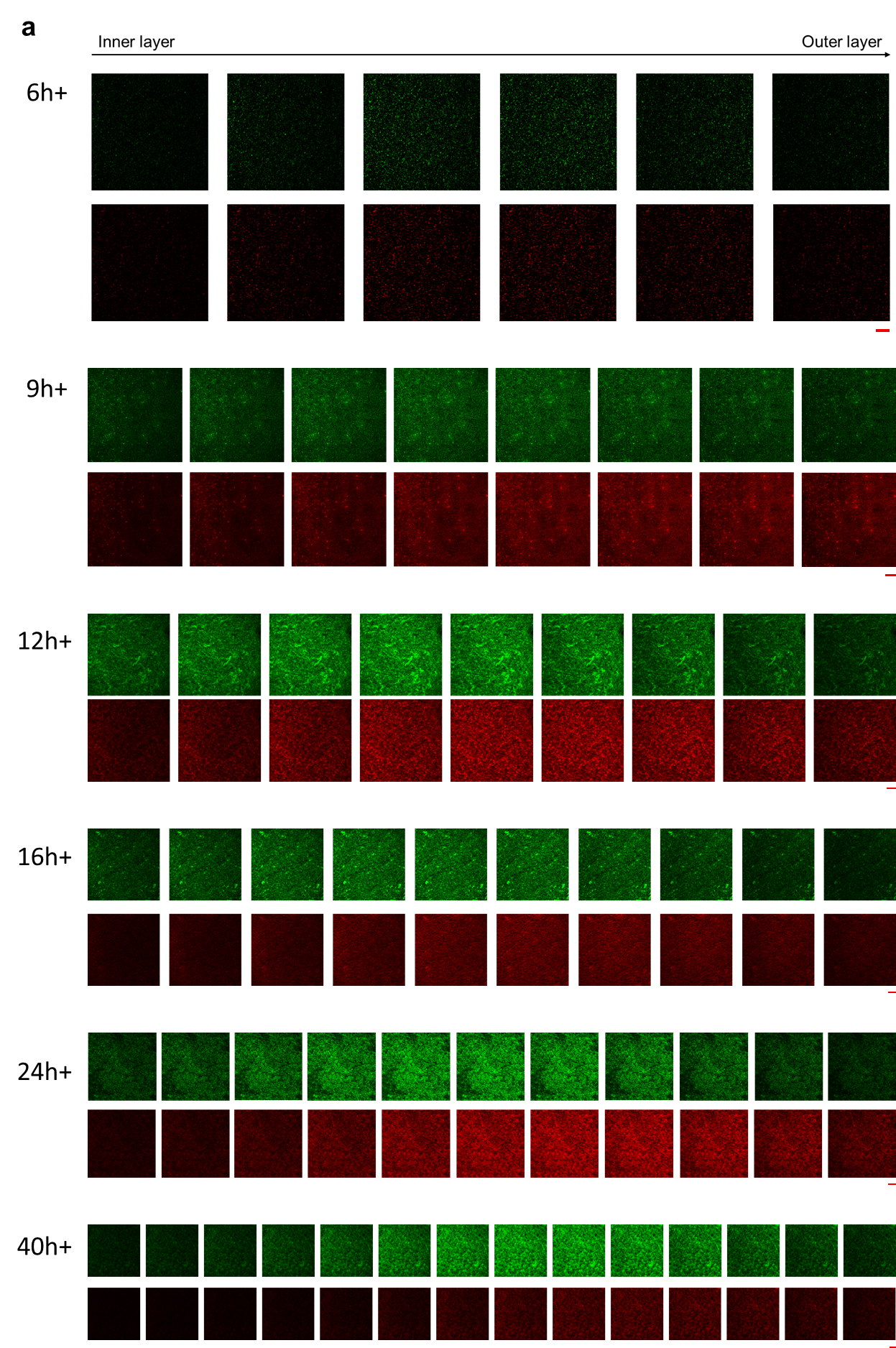


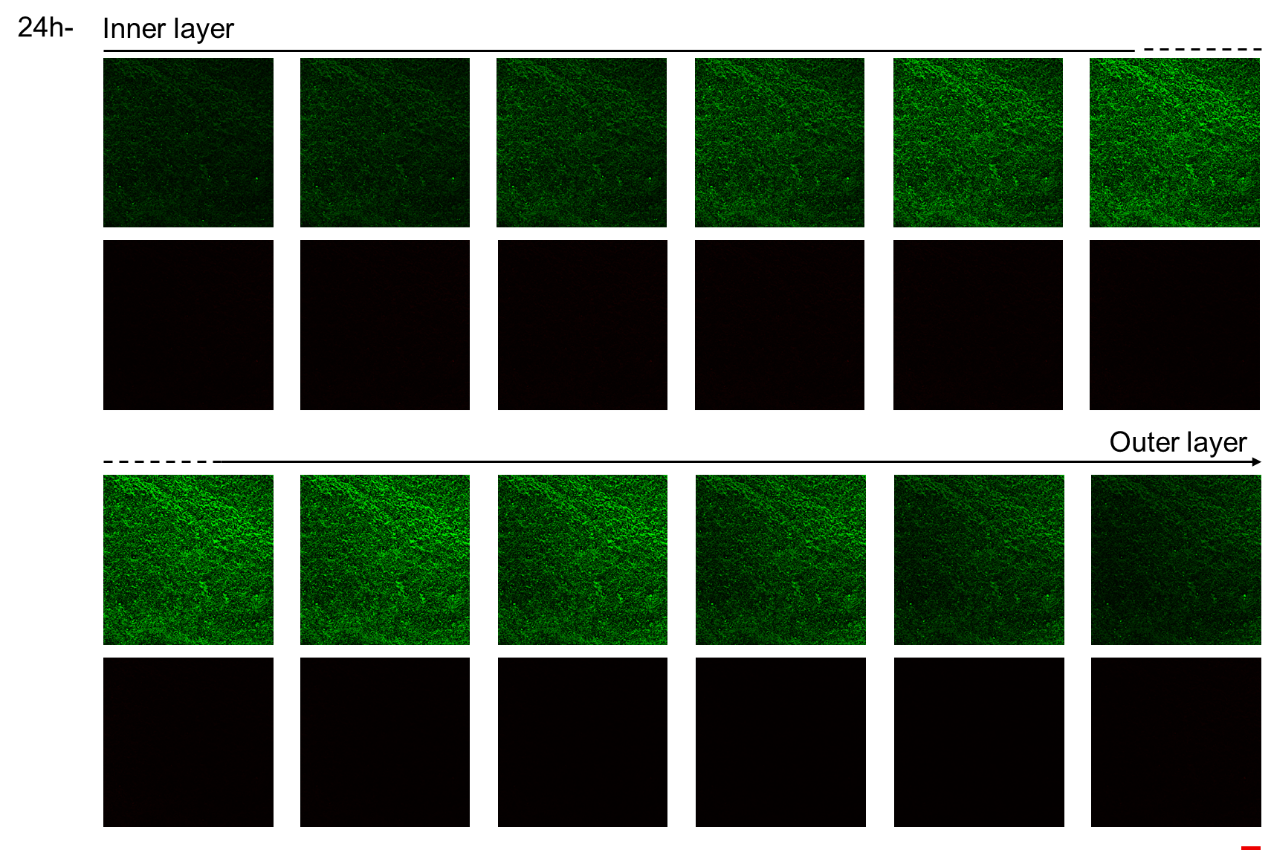


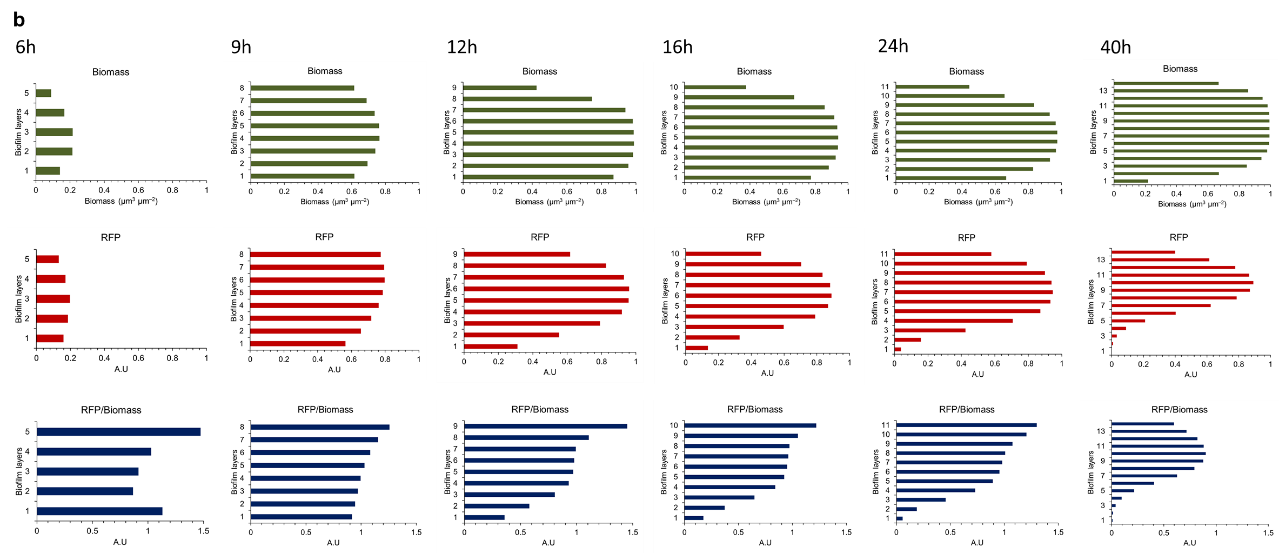


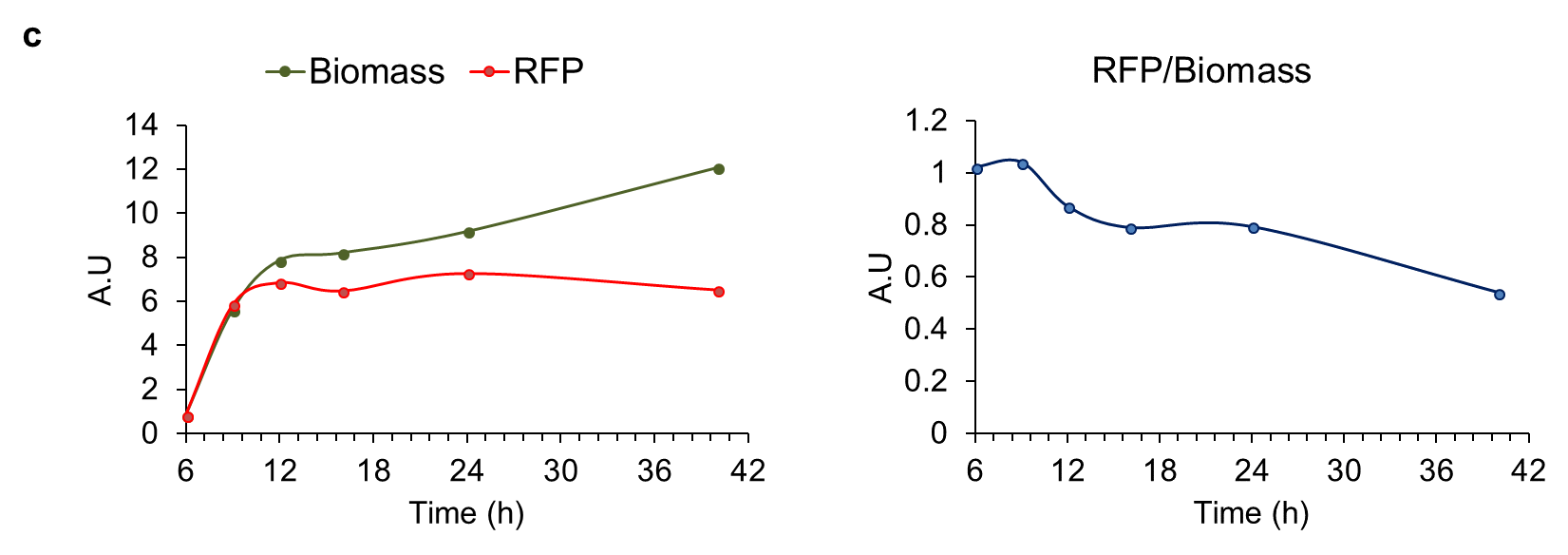


**Figure S3:** **Heterogenous gene (RFP) expression in the biofilm. Related to Figure 2.**

To study heterogenous gene expression in biofilm which induced by externally added inducer, aTc inducible RFP expression plasmid pBbE2k-pTet-RFP and constitutive GFP expression plasmid pD2-dcas9-J23101-GFP (GFP was used to indicate the biomass) were co-transformed into *E. coli* MG1655. The biofilms were grown to 6^th^, 9^th^, 12^th^, 16^th^, 24^th^ and 40^th^ hour, followed by 100 nM aTc induction for 4 hours to induce RFP expression. For the control that grew to 24^th^ hours, no inducer was added, and biofilm was harvested at 28^th^ hour. Biofilm was scanned by CLSM sectioning to obtain images at different biofilm layers. Our hypothesis was that less RFP would be expressed in the inner layer of the biofilm, because less oxygen, nutrients and aTc could diffuse into the inner layer of the biofilm.

a) Confocal images of different biofilm layers. Green colour stands for the biomass and red colour is the RFP. The images show that RFP expression decreased at the inner layers. RFP at the inner lay became less when biofilm grows thicker.

b) Total RFP expression gives an indication of overall cell activity within the biofilm. More RFP means more cells are induced and accessible to aTc inducer. RFP/Biomass gives an indication of activity per cell or active cell versus total cells. RFP in the inner layer at 24 and 40 hours decreased faster as compared with these from 6 to 16 hours. This is likely due to the diffusion barrier established by the thick biofilm, the inner layer gets less access to the inducer, nutrients and oxygen.

c) RFP/Biomass was relatively higher at 9 hours and 12 hours, and it reached steady value after 16 hours. Further, the overall RFP has also started to saturate as well after 16 hours. Besides, the overall RFP in 40 hours biofilm decreased, although the biomass increased as compared to 24 hours biofilm.

From the current observation, too thick a biofilm led to a decrease in productivity while too thin a biofilm could not achieve good yield. As a conclusion, biofilm should operate within certain range of thickness in order to achieve good RFP expression. Therefore, maintaining the biofilm at certain thickness is useful. Scale bars = 50 um.


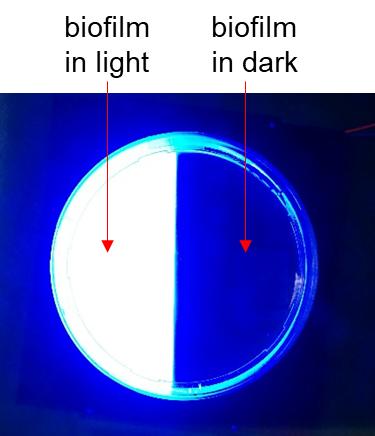


**Figure S4: The setup used for spatially controlling biofilm using blue light. The light system setup for spatial control of biofilm thickness. Related to Figure 3.**

Blue light was shone from bottom, while half of the area was covered by a semicircle photomask. Only half of the area on the microscope slide would be exposed to light.


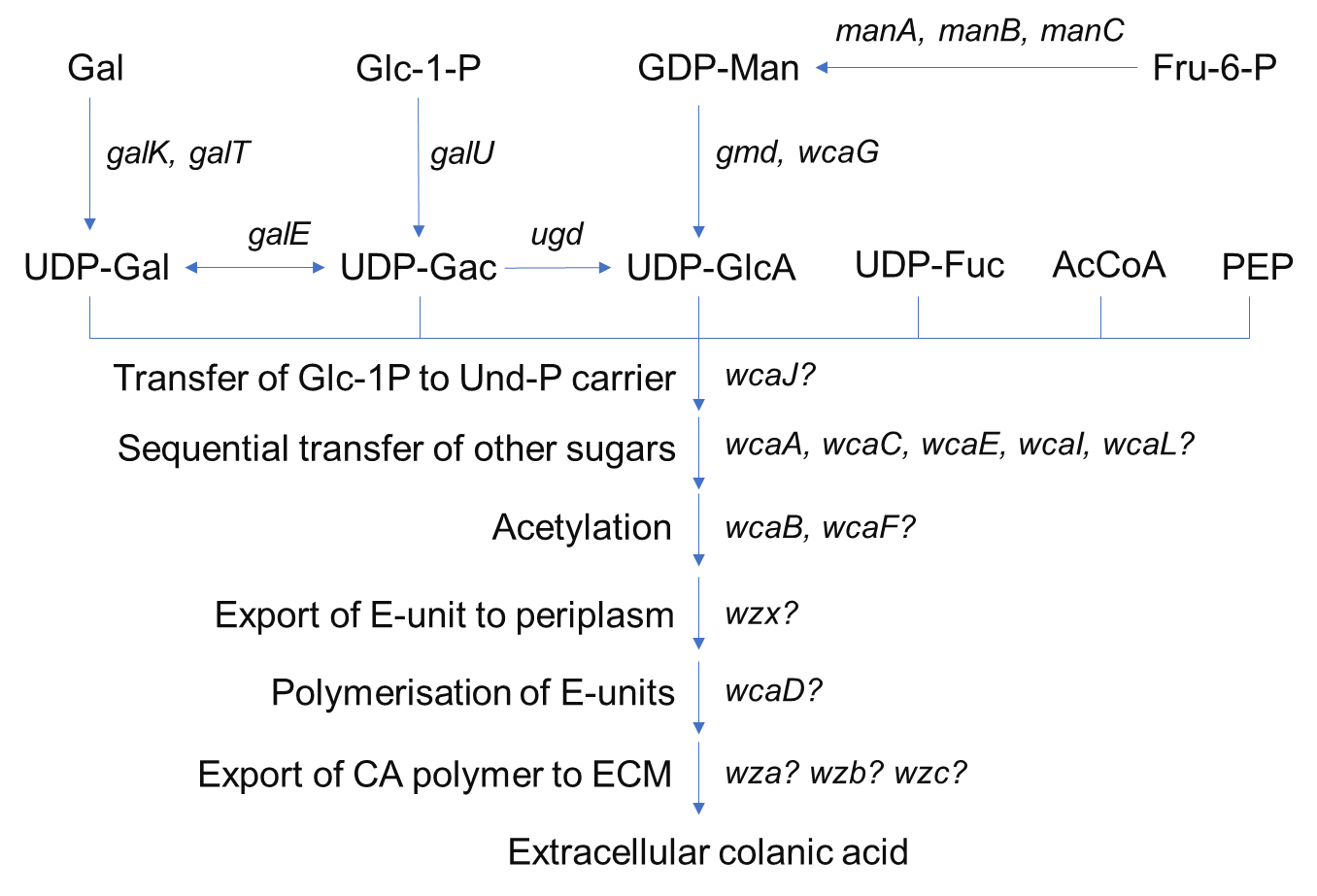


**Figure S5: Proposed pathway for CA synthesis in E. coli.**

Genes that encode the enzyme for bioconversion in each step are shown. Question mark indicates the gene’s functions is putative. With reference to ^1^.

# Table 1

| **Plasmids** | **Strain** | **Description** |
| --- | --- | --- |
| pBbE2k-pTet-RFP | *E. coli* MG1655 | aTc inducible RFP expression plasmid |
| pD2-dcas9-J23101-GFP | *E. coli* MG1655 | Constitutive dCas9 and GFP expression plasmid |
| pBbE2k-pTet-gRNAwcaF_159_ | *E. coli* MG1655 | aTc inducible gRNA expression plasmid |
| pBbE2k-pTet-gRNA_wcaF159_-pBAD-bdcA_E50Q | *E. coli* MG1655 | aTc inducible gRNA and arabinose inducible bdcA_E50Q expression plasmid |
| Brep-gRNA_wcaF159_ | *E. coli* MG1655 | Blue light repressible gRNA expression plasmid |
| pBbE8k-pBAD-bdcA_E50Q-J23101-mCherry | E. coli MG1655 | Arabinose inducible bdcA_E50Q expression plasmid |

| **Primers** | **Sequence (5'-3')** |
| --- | --- |
| BLRS 102-RFP FWD | acgtgctcgctcgatgcgatgtttcgcttggtggtcgaat |
| BLRS 102-RFP REV | GCTCTAAAACATTTTTGCTCCGAATAAACGaGATCCGCTAGCACAGTACA |
| pBbP6k-wcaF159 FWD | TAGCGGATCtCGTTTATTCGGAGCAAAAATGTTTTAGAGCTAGAAATAGC |
| pBbP6k-wcaF159 REV | AGGACTGAGCTAGCCGTCAAtataaacgcagaaaggccca |
| El222 FWD | tgggcctttctgcgtttataTTGACGGCTAGCTCAGTCCT |
| El222 REV | atttgatgcctggagatcctTTAGATTCCGGCTTCGACGG |
| pBbE8k FWD | CCGTCGAAGCCGGAATCTAAaggatctccaggcatcaaataaaacg |
| pBbE8k REV | attcgaccaccaagcgaaacatcgcatcgagcgagcacgt |
| wcaF159 R | ATTTTTGCTCCGAATAAACGgtgctcagtatcttgttatc |
| wcaF159 F | CGTTTATTCGGAGCAAAAATGTTTTAGAGCTAGAAATAGC |
| pBbE2k-RFP REV | ATTTTTGCTCCGAATAAACGagatcttttgaattcttttctctatcactga |
| E2k-159-FWD | atctCGTTTATTCGGAGCAAAAATGTTTTAGAGCTAGAAATAGC |
| pBbP6k-gRNA(F)-wcaF159 REV | catggctgatgcaatgcggcggctgcatacgcttgatccg |
| pBbE2k-RFP FWD | cggatcaagcgtatgcagccgccgcattgcatcagccatg |
| pBbE8k-E50Q FWD (1) | tggtcgaatgggcaggtagccggatcaagcgtatgcagcc |
| pBbE8k-E50Q REV (1) | tcggaattgccagctggggcataaccgtattaccgcctttga |
| pBbE2k-wcaF159 FWD | aaaggcggtaatacggttatgccccagctggcaattccga |
| pBbE2k-wcaF159 REV | ggccttttgctggccttttggcctttgagtgagctgataccg |
| pBbE8k-E50Q FWD (2) | gtatcagctcactcaaaggccaaaaggccagcaaaaggccaggaa |
| pBbE8k-E50Q REV (2) | ggctgcatacgcttgatccggctacctgcccattcgacca |
|  |  |
| gRNAwaF_159_ targeting region (highlighted sequence is the gRNA targeting site) | ATGCAAGATTTAAGCGGTTTCTCGGTGCCGAAAGGGTTCCGGGGCGGCAACGCTATTAAAGTGCAATTATGGTGGGCAGTACAGGCAACAATATTTGCCTGGTCGCCACAAGTATTGTATCGCTGGCGGGCTTTTTTATTACGTTTATTCGGAGCAAAAATAGGAAAAAACGTAGTTATTCGTCCGTCAGTAAAAATTACCTATCCGTGGAAATTAACCTTAGGTGATTACGCGTGGGTCGGCGATGACGTCAATTTATATACCCTCGGTGAAATAACCATTGGCGCACATTCGGTGATATCGCAAAAAAGTTATTTATGCACCGGTAGCCACGACCATGCAAGTCAACATTTCACCATTAACGCCACGCCTATTGTGATTGGCGAGAAATGCTGGCTGGCAACCGATGTCTTTGTTGCCCCAGGCGTCACAATCGGCGACGGCACCGTCGTGGGTGCACGAAGCAGTGTTTTTAAATCGCTTCCGGCAAATGTGGTTTGCCGGGGGAATCCCGCAGTGGTGATACGCGAACGCGTTGAAACTGAATAA |

# Reference

1 Stevenson, G., Andrianopoulos, K., Hobbs, M. & Reeves, P. R. Organization of the Escherichia coli K-12 gene cluster responsible for production of the extracellular polysaccharide colanic acid. *Journal of bacteriology* **178**, 4885-4893, doi:10.1128/jb.178.16.4885-4893.1996 (1996).
